# Congenital factor VII deficiency in Iraqi children (Single Centre Experience)

**DOI:** 10.12669/pjms.36.2.666

**Published:** 2020

**Authors:** Ali Ahmed Khudhair, Afrah Abdul-Mahdi Salih, Ausama Jamal Kadhum

**Affiliations:** 1 Dr. Ali Ahmed Khudhair, C.A.B.P, FICMS (PED). Department of Pediatrics, Children Welfare Teaching Hospital, Baghdad, Iraq; 2 Dr. Afrah Abdul-Mahdi Salih, C.A.B.P, MRCPH. Department of Pediatrics, Children Welfare Teaching Hospital, Baghdad, Iraq; 3 Dr. Ausama Jamal Kadhum, C.A.B.P. Department of Pediatrics, Children Welfare Teaching Hospital, Baghdad, Iraq

**Keywords:** Bleeding disorder, Congenital factor VII deficiency

## Abstract

**Background and Objective:**

Factor VII (FVII) deficiency is probably one of the most common of the rare autosomal recessive coagulation disorders, with an estimated prevalence of l: 500000. All age groups can be affected with FVII deficiency. This study aimed to describe the demographic parameters, symptomatology, hemostatic values and the outcome of FVII deficiency.

**Methods:**

This is a retrospective descriptive study of patients with congenital FVII deficiency over a period of seven years from (August 2008 to August 2015). The data were collected by reviewing the files for each patient diagnosed with FVII deficiency. Surgical interventions, complications and follow up visits were recorded.

**Results:**

Twenty-four patients were included in this study, 17 females and seven males, below one year was the most common age at presentation. More than half of patients (58.3%) were diagnosed within six months of symptoms onset. The majority of patients had severe phenotype. The most common symptom was epitaxis (41.7%). Five out of 10 patients with FVII level < 1% have either mild to moderate phenotype of the disease without complications; while six out of 14 patients with FVII > 1% had at least one episode of severe bleeding. Three patients had hepatitis C; all were treated by blood products before the introduction of recombinant FVII in Iraq. The outcome of most patients (75%) was normal without complications at time of study.

**Conclusion:**

Clinical manifestations of FVII deficiency are variable and they are not necessarily correlated to the FVII level.

## INTRODUCTION

Factor VII (FVII) is Vitamin-K dependent glycoprotein that is synthesized in the liver and circulates in blood as an inactive zymogene. The FVII gene is situated on the long arm of chromosome 13q34 OMIM: 227500.

FVII levels are modified by a number of environmental and genetic variables. Among the environmental influences shown to correlate positively with FVII levels are age, dietary fat intake, levels of plasma triglyceride and cholesterol, gender, obesity and the presence of diabetes.^1^

Factor VII, in its activated form (factor VIIa), participates in the initiation of coagulation via the extrinsic pathway in association with tissue factor, an integral membrane protein that is exposed to the circulation upon vascular injury or stimulation of monocytes and endothelial cells.^2^

### Factor VII deficiency

It was first defined in 1951 by Alexander et al and was called (Alexander´s disease). With an estimated prevalence of l: 500000. Inherited FVII deficiency is probably one of the most common of the rare, autosomal recessive coagulation disorders.However, the true prevalence is still not known. The 2010 World Federation of Hemophilia Annual Global Survey comprising data from 106 countries reported a total of 4938 persons with FVII deficiency, a number that represents 28% of all RBDs, excluding platelet disorders.^2^

The hemorrhagic predisposition in affected patients is highly variable and correlates poorly with plasma factor VII activity levels.^3^ In recent years, at least 30 different mutations in the factor VII structural gene have been identified in patients with factor VII deficiency.^4^

Moderate and mild bleeding phenotypes are usually defined as a presence of >3 and 1–2 mucocutaneous bleeding symptoms in an individual patient, respectively, while gastrointestinal, central nervous system, muscle, and joint bleeding define a severe phenotype.

### Clinical manifestations

Bleeding in patients affected by inherited FVII deficiency is extremely heterogeneous concerning both sites and severity. Early reports suggested that intracranial bleeding is a common symptom of severe FVII deficiency.^5^

Bleeding in the severest cases starts within the first 6 months of life, On the other hand, patients with FVIIc levels greater than 5% have been reported to have a personal history of severe bleeding symptoms.^6^ In mild bleeding, mimicking the clinical picture of a platelet disorder, epistaxis and easy bruising; gum bleeding being the most frequent type of hemorrhage. Haemarthrosis usually arises when infants start to crawl or walk and when the same is a prominent element in the clinical phenotype because of frequent recurrences, it is almost invariably associated with very low FVII levels.^5^ In mildly and moderately affected patients, symptoms include one or more bleeding symptoms other than intracranial, gastrointestinal and joint bleeding.

Such patients are diagnosed with an inconstant delay until traumatic events or surgery occurs. As in the other autosomally inherited congenital bleeding disorders menorrhagia is a very frequent type of bleeding in women with FVII deficiency, accounting for two-thirds of the bleeding incidence among women aged 10-50 years with a peak prevalence in teenagers.

### Diagnosis

Homeostasis screening tests in patients with factor VII deficiency reveal a prolonged PT with a normal PTT and a normal TT. The diagnosis is confirmed by specific FVII assay using a one-stage clotting assay.

Heterozygote typically have normal PTs and are identified only after specific factor VII assays are performed in family members of patients with homozygous or compound heterozygous factor VII deficiency.

Factor VII levels in homozygotes and compound heterozygotes are usually below 3 IU/dL. The levels in heterozygotes usually range between 15 IU/dL and 50 IU/dL. Based on the observations in the FVII knock-out mouse, it is believed that a complete absence of FVII is incompatible with life.^7^

### Treatment of FVII deficiency

Fresh frozen plasma (FFP), prothrombin-complex concentrates (PCC), activated prothrombin-complex concentrates PCC (aPCC), plasma-derived human FVII (pdFVII) and recombinant activated FVII (rFVIIa) may be used for bleeding episodes in FVII deficiency. Recombinant FVIIa is a recombinant product that can be easily dose adjusted and administered without any serious side effects. For these reasons, its use has been preferred mostly.^5^ The aim of the study was to describe the demographic parameters, clinical presentation, haemostatic values and outcome in patients with congenital factor VII deficiency.

## METHODS

This is retrospective descriptive study of patients who were diagnosed with congenital FVII deficiency in Hemophilia Ward in Children Welfare Teaching Hospital/Medical City in Baghdad over a period of seven years from August 2008 to August 2015. The patients were diagnosed by having prolonged PT with normal PTT and confirmation based on low FVII assay < (50%). Patients with acquired FVII deficiency were excluded from the study.

The data were collected by reviewing the files for each patient diagnosed with FVII deficiency. Surgical interventions, complications and follow up visits were recorded. Before August 2008 patients were treated by fresh frozen plasma, and after that date recombinant FVII had been introduced to our country. All the laboratory tests were performed at the coagulation lab. Of children welfare teaching hospital. The normal range of FVII level is (50-150%).Patients with a congenital FVII deficiency have factor VII level of less <50%. The concentration measured may vary according to thromboplastin used.^8^

Patients recognized as sever phenotype if they had at least one of the following symptoms: gastrointestinal (GI), central nervous system (CNS) bleedings or haemarthrosis with or without other bleeds, and moderate phenotype if they had three or more symptoms with exception of GI, CNS bleeding or haemoarthrosis,and those who had one or two symptoms with the exception of GI, CNS bleeding or haemoarthrosis are considered mild phenotype.^9^

### Statistical analysis

The data were analyzed using Statistical Package for Social Sciences (SPSS) version 20. The level of significance in this study was of 0.05.

## RESULTS

A total number of 24 FVII deficient patients were identified during this study period; there were 17 (70.8%) females and 7 (29.2%) males. The age of diagnosis was < 1 year in 9(37%) patients, 1-5 years in 3(12.5%) patients, 5-10 years in 5(20.8%) patients and > 10 years in 7 (29.2%) patients, Table-I.

**Table-I:** Demographic parameters of FVII deficient patients, n=24.

Variables		Number	Percent
Sex	Male	7	29.2%
	Female	17	70.8%
Age at diagnosis	< 1 year	9	37.50%
	1 - <5 years	3	12.50%
	5 - 10 years	5	20.80%
	> 10 years	7	29.20%
Factor VII level (%)	<1%	10	41.7%
	1% - 5%	4	16.7%
	> 5%	10	41.7%
Family history of F VII def.	No	13	54.2%
	Yes	11	45.8%
Severity	Mild	10	41.7%
	Moderate	3	12.5%
	Severe	11	45.8%
Outcome	Normal	18	75.0%
	Arthropathy	3	12.5%
	Cerebral palsy	2	8.3%
	Died	1	4.2%

The level of the FVII were as the following, < 1% in 10(41.7%) patients, 1%-5% in 4(16.7%) patients and > 5%in 10(41.7%) patients Table-I. Of the 24 patients included in this study, the family history of FVII deficiency was positive in 11(45.8%) patients with rate of consanguinity among parents of the patients was 83.3% Table-I.

Parental consanguinity (far from 3rd degree) was presents in all (11) patients with severe phenotype of the disease and it was present in 9 out of 13 patients with mild to moderate disease (p-value0.044) so there is a significant correlation between severity and consanguinity Table-II.

**Table II:** Relation between parents’ consanguinity of the included patients and severity of factor VII deficiency, n=24.

Severity	Consanguinity No. (%)		Total (%)

	Yes	No	
Mild to moderate	9 (45)	4 (100)	13 (54.2)
Severe	11 (55)	0 (0)	11 (45.8)

Total	20 (100)	4 (100)	24 (100)

Fisher’s exact test, p-value = 0.044* (significant at 0.05 level)

According to clinical phenotyping in literature review classification of FVII deficiency in this study was as the following: mild in 10(41.7%) patients, moderate in 3(12.5%) patients and severe phenotype in 11(45%) patients Table-I.

Hepatitis C (HCV) was positive in 3(12.5%) patients and all of them were treated with FFP (fresh frozen plasma) and two of them received blood transfusion and that was before the introduction of r FVII in Iraq, and after the introduction of this factor at August 2008 no more use for FFP as a treatment option.

The most common presenting symptoms are bleedings from different sites and most common one is the mucocutaneous bleeding (21%), on the other hand three patients presented with menorrhagia and three with postsurgical bleeding (two of them after tonsillectomy and one after circumcision).

The most common symptoms of FVII deficiency are those related to the skin and mucous membrane, epistaxis (41.7%) and easy bruising (33.3%). The percentage of menorrhagia frequency is 60% (3 out of 5 patients) when it is adjusted for gender (female) and age (older than 12 years which is the age of youngest female patient who have menarche in this study).

During our study period it was found that 5(50%) out of 10 patients with FVII level <1% have evidence of at least one episodes of severe bleeding (gastrointestinal, intracranial bleeding and hemoarthrosis), while the other 5 (50%) of the patients had have either mild or moderate bleeding episodes. For those patients with FVII level >1%, 6(42.8%) out of 14 patients had severe phenotype and 8(57.1%) had mild to moderate phenotype. Which indicate that the severity of bleeding dose not correlate with the level of FVII coagulant activity measured in plasma Table-III.

**Table-III:** The relation between FVII level in factor FVII deficient patients and the phenotyping severity of the disease.

FVII lab level	Phenotyping Severity No. (%)		Total (n=24)

	Mild to Moderate	Sever	
<1%	5 (50)	5 (50)	10 (100)
>1%	8 (57.1)	6 (42.8)	14 (100)

Total	13 (100)	11 (100)	24 (100)

The percentage of developing complications (arthropathy, cerebral palsy and death) in patients with FVII deficiency was 25% (6 out of 24patients). It was found that 5(45%) of 11 patients with severe phenotype FVII deficiency had complications and 1(7.7%) of 13 patients with mild to moderate phenotype of FVII deficiency developed complication (p-value=0.029) so there is significant correlation between severity and complication Table-IV.

**Table IV:** Relation between presence of complications among the included patients and severity of factor VII deficiency, n=24.

Complications	Severity No.(%)		Total No.%

	Mild to moderate	Sever	
Absent	12 (92.3)	6 (54.5)	18 (75)
Present	1 (7.7)	5 (45.5)	6 (25)

Total	13 (100)	11 (100)	24 (100)

Fisher’s exact test, p-value = 0.029* (significant at 0.05 level)

The outcome of FVII deficient patients were as the following; 18(75%) patients were normal at time of study, 3(12.5%) patients developed arthropathy, 2(8.3%) patients had Cerebral palsy and one patient died due to intracranial bleeding. no case reported for FVII inhibitor in this study Table-I.

## DISCUSSION

In this study the females were found to be more affected than males and this agree with Tripathi et al. 2019^8^, but disagree with Salcioglu et al. 2012^10^ which found (65.8%) males and (34.2%) females.

More than half of the patients (58.3%) were diagnosed within six months of onset of bleeding symptoms; this is compatable to Mariani G et al. 2008^5^ who showed that (50.5%) of patients with FVII were diagnosed within 6 months of the presentation, the delay in the diagnosis may be due to treatment of the patients with blood products that mask the underlying coagulopathy.

The level of FVII in affected patient in this study was compatible to Alam MM et al. 2015^11^ Who reported that FVII level was as the following <1%, 1-5% and > 5% in 41%, 24% and 35% patient respectively. And that is compatiple also with Tripathi et al. 2019^8^: 50% of cases had factor VII levels less than 1% and 25% of cases the level was between 2% and 10%.

In this study the majority of the patients had severe phenotype and this disagree with Salcioglu Z et al. 2012^10^_,_ Who found severe phenotype was only (23%) and this may be explained by shortage of (r FVIIa) which make the patients developed recurrent bleeding episodes mainly hemarthrosis and because of asymptomatic patient could not diagnosed in this study due to lack of facilities in Iraq.

The frequency of Central nervous system bleeding was 8.3% and this agree with Napolitano et al 2017^12^_,_ which found that 10% to 15% exhibit potentially life- or limb-threatening hemorrhages (CNS). Another severe hemorrhage type in this study is GI bleeding (29.1%); and this compatible to Mariani G et al. 2008^5^ who reported its frequency was (28.2%) of patients, while in Salcioglu Z et al. 2012^10^_._ The frequency of GI bleeding episodes was found to be 7.1%. On other hand hemoarthrosis, its frequency (8.3%) comparable to that reported by Alam M et al. 2015^11^_,_ who reported (6%) of patients, While Napolitano et al. 2017^12^ reported recurrent hemarthrosis as (19%).

In this study we found that 50% (5 out of 10) patients with FVII level < 1% have mild to moderate phenotype of the disease without complications while 42.8% (6out of 14) patients with FVII level > 1% had at least one episode of severe bleeding (GI, ICB and hemarthrosis) and this is matched with Alam MM et al 2015^11^ who reported that 55% of the patients with FVII level <1% were either asymptomatic or mildly symptomatic, where as 53% patients with FVII > 5% were affected by severe symptoms and this reflect the poor correlation between the level of FVII and the severity of bleeding. And this agree in general with Napolitano et al 2017.^12^

## CONCLUSION

Clinical manifestations of FVII deficiency are variable and they are not necessarily correlated to the FVII level. Epistaxis and easy bruising are the most common presenting symptoms for FVII deficiency, FVII deficient patients who treated with blood or blood product are at high risk for acquisition of hepatitis C virus (HCV), while this complication can be eliminated by the usage of r FVIIa for treatment of FVII deficiency. Most of our patients were diagnosed within six months of the symptoms onset, most of them are normal without complications or chronic disabilities.

### Author`s Contribution:

**AAS** conceived designed and final approval of manuscript, is responsible for integrity of research.

**AAK** did data collection, statistical analysis and manuscript writing.

**AJK** did review & editing of manuscript.
